# The HubBLe trial: haemorrhoidal artery ligation (HAL) versus rubber band ligation (RBL) for haemorrhoids

**DOI:** 10.1186/1471-230X-12-153

**Published:** 2012-10-25

**Authors:** Jim Tiernan, Daniel Hind, Angus Watson, Allan J Wailoo, Michael Bradburn, Neil Shephard, Katie Biggs, Steven Brown

**Affiliations:** 1 CRUK Clinical Research Fellow, St James’ University Hospital, Leeds, LS9 7TF, UK; 2 Sheffield Clinical Trials Research Unit, School of Health and Related Research, Regent Court, 30 Regent Street, Sheffield, S1 4DA, UK; 3 Department of Surgery, Raigmore Hospital, Inverness, IV2 3UJ, UK; 4 Health Economics and Decision Sciences, School of Health and Related Research, Sheffield, UK; 5 Northern General Hospital, Sheffield, S5 7AU, UK

**Keywords:** Haemorrhoids, Rubber band ligation, Haemorrhoidal artery ligation, Surgical randomised controlled trial

## Abstract

**Background:**

Haemorrhoids (piles) are a very common condition seen in surgical clinics. After exclusion of more sinister causes of haemorrhoidal symptoms (rectal bleeding, perianal irritation and prolapse), the best option for treatment depends upon persistence and severity of the symptoms. Minor symptoms often respond to conservative treatment such as dietary fibre and reassurance. For more severe symptoms treatment such as rubber band ligation may be therapeutic and is a very commonly performed procedure in the surgical outpatient setting. Surgery is usually reserved for those who have more severe symptoms, as well as those who do not respond to non-operative therapy; surgical techniques include haemorrhoidectomy and haemorrhoidopexy. More recently, haemorrhoidal artery ligation has been introduced as a minimally invasive, non destructive surgical option.

There are substantial data in the literature concerning efficacy and safety of 'rubber band ligation including multiple comparisons with other interventions, though there are no studies comparing it to haemorrhoidal artery ligation. A recent overview has been carried out by the National Institute for Health and Clinical Excellence which concludes that current evidence shows haemorrhoidal artery ligation to be a safe alternative to haemorrhoidectomy and haemorrhoidopexy though it also highlights the lack of good quality data as evidence for the advantages of the technique.

**Methods/design:**

The aim of this study is to establish the clinical effectiveness and cost effectiveness of haemorrhoidal artery ligation compared with conventional rubber band ligation in the treatment of people with symptomatic second or third degree (Grade II or Grade III) haemorrhoids.

Design: A multi-centre, parallel group randomised controlled trial.

Outcomes: The primary outcome is patient-reported symptom recurrence twelve months following the intervention. Secondary outcome measures relate to symptoms, complications, health resource use, health related quality of life and cost effectiveness following the intervention.

Participants: 350 patients with grade II or grade III haemorrhoids will be recruited in surgical departments in up to 14 NHS hospitals.

Randomisation: A multi-centre, parallel group randomised controlled trial. Block randomisation by centre will be used, with 175 participants randomised to each group.

**Discussion:**

The results of the research will help inform future practice for the treatment of grade II and III haemorrhoids.

**Trial Registration:**

ISRCTN41394716

## Background

Haemorrhoidal tissue, which forms the ‘anal cushions’, is a normal component of the anal canal and is composed predominantly of vascular tissue, supported by smooth muscle and connective tissue. Haemorrhoids result from enlargement of the haemorrhoidal plexus and pathological changes in the anal cushions. They are common, affecting as many as 1 in 3 of the population [1]. Approximately 23,000 haemorrhoidal operations were carried out in England in 2004/5 [2] and the prevalence may be even higher in professionally active people. Repeated visits to hospital for therapy represent a significant disruption to the personal and working lives for this population in particular.

Treatment is dictated by the degree of symptoms and the degree of prolapse, and ranges from dietary advice to rubber band ligation (RBL) in the outpatient department, to an operation under general or regional anaesthetic. Although RBL is cheap, it has a high recurrence rate and patients often require further visits to the outpatient department for repeat banding before exploring surgical options [3]. Although there are some variations (such as ligasure haemorrhoidectomy), surgery is commonly traditional "open" haemorrhoidectomy (OH) or a stapled haemorrhoidopexy (SH); both require an anaesthetic. OH is associated with considerable post-operative discomfort, sometimes necessitating overnight hospital stay and a delay in return to normal activity, but has a low recurrence rate; SH has a slightly higher recurrence rate but is carried out as a day case and patients return to normal activity more quickly [4]. An alternative treatment is haemorrhoidal artery ligation (HAL), which also requires an anaesthetic, but is thought to enable even quicker return to normal activity. Recurrence rates are reportedly similar to SH but complication rates are lower [5].

There are substantial data in the literature concerning efficacy and safety of RBL including multiple comparisons with other interventions [6-12]. Recurrence varies from 11% to over 50%. This broad range probably reflects the definition of recurrence (patient symptoms or clinical appearance), the grade of haemorrhoids treated (grade I know prolapse; grade II spontaneously reducible prolapse; grade III prolapse requiring manual reduction; and grade IV un-reducible prolapse), the number of treatments and/or the intensity and length of follow up. In most studies, the incidence of recurrence is more than 30% and appears greatest for grade III haemorrhoids. Pain is common for a few hours following RBL and occasionally patients experience pain so severe as to require admission to hospital (around 1% [3]), bleeding (3-4%, sometimes necessitating further treatment [10]) and vaso-vagal symptoms (3% [13]). There have also been rare incidences of blood transfusion [14,15] and severe pelvic sepsis with a few instances leading death [13]. Recurrences can be treated by re-banding or by surgical intervention.

Although HAL requires an anaesthetic, evidence suggests a recovery similar to RBL but an effectiveness that approaches the more intensive surgical options. The substantial data concerning effectiveness includes one recent systematic review [5], three Randomised Control Trials (RCTs) [16-18], one non-randomised trial [19] and over 20 case series. A recent overview has been carried out by the National Institute for Health and Clinical Excellence (NICE), which concludes that current evidence shows it to be a safe alternative to OH or SH [20], this is summarised below.

In terms of efficacy, studies with more than 1 year follow up suggest bleeding, pain on defecation and prolapse (surrogates of recurrent symptoms) in 10%, 9% and 11% of patients respectively

Regarding safety, post-operative haemorrhage requiring intervention (readmission, transfusion, reoperation or correction of coagulopathy) was reported in less than 1.2%, haemorrhoidal thrombosis was seen in less than 3.5% and fissure formation in less than 2.1%

The data from the three RCTs comparing HAL with SH and OH is difficult to combine, but efficacy seems similar for all procedures, with OH perhaps being superior in treating prolapse, although it is unclear if a "pexy" stitch was used in the HAL cases to reduce prolapse. OH appears to lead to the most post-operative pain and longest recovery. There are conflicting results as to whether the HAL technique results in less pain compared with SH. Complications were also more frequent in the OH group but occurred at a similar frequency when SH and HAL were employed.

Both the systematic review and the NICE overview highlight the lack of good quality data as evidence for the advantages of the technique; most data is from case series. Even the RCTs have significant methodological drawbacks that make them subject to selection, performance, attrition and detection bias. Indeed none of the studies are powered to reach any meaningful conclusion. There are no existing studies that compare HAL with RBL.

## Methods/design

The trial will be co-ordinated from the Clinical Trials Research Unit (CTRU) in Sheffield School of Health and Related Research (ScHARR). Delegated study staff located at individual centres will identify and consent potential participants. Potential participants will fall into three groups:

1. Patients presenting to the surgical outpatient clinic (SOPC) with symptomatic haemorrhoids that do not require further tests. This group will be identified by the clinical team from the GP referral letter and a patient information sheet sent to them prior to their clinic appointment. If they are willing to participate they will be consented and randomised when they attend the appointment.

2. Patients presenting to the SOPC with symptomatic haemorrhoids that require further tests to exclude other diagnoses. This group will be identified by the clinician at the clinic appointment and given a patient information sheet. They will undergo the necessary outpatient tests (usually endoscopy) and if negative (i.e. the symptoms are due to haemorrhoids) they will be contacted by the research nurse prior to attending their follow-up clinic appointment. They will then be randomised and consented when they re-attend the clinic.

3. Patients who return to SOPC following one unsuccessful RBL. They will be identified by the clinician at their first clinic appointment (when they have RBL) and given a patient information sheet. They will be contacted prior to a follow-up appointment (usually six weeks after treatment) by a research nurse. If they remain symptomatic and are willing to participate, they will be consented and randomised when they re-attend.

Thus, in each group, there is opportunity to provide the patient information sheet prior to a clinic appointment. Patients with investigations excluding pathologies other than haemorrhoids, and all those who have undergone rubber band ligation, will be contacted by the research nurse before the planned follow up clinic to ascertain whether they meet entry criteria and are interested in entering the trial. They will then be seen by the consultant and research nurse in clinic where recruitment and randomisation will take place.

After consent, participants will be individually randomised to HAL or RBL in equal proportion at all centres using a remote, web-based randomisation system.

Data will be collected to establish which patients have further treatment for recurrent symptoms or complications following their initial procedure. This will be achieved at the six week clinic visit following the intervention and by interrogating hospital records, asking the patients’ consultants, writing to patients’ GPs and questioning the patient via telephone interview at 12 months. Due to appointment availability the six week clinic visit may actually vary from four to twelve weeks following the intervention; this window is seen as clinically relevant.

### Participants

The target population will be patients referred to collaborating centres for treatment of haemorrhoids. 350 participants will be recruited from surgical departments in up to 14 NHs hospitals. Adults aged 18 years or over with symptomatic second or third degree haemorrhoids, either presenting for the first time or after one failure of RBL will be eligible to take part in the research.

### Exclusion criteria

Patients with certain pre-existing medical conditions will be excluded: patients with known perianal sepsis, inflammatory bowel disease, colorectal malignancy, pre-existing sphincter injury, or an immunodeficiency. Pregnant women and patients that are unable to have general or spinal anaesthetic are excluded as well as patients currently taking certain medication: Warfarin, Clopidogrel and Nicorandil.

Patients that are unable to give full informed consent (this may be due to mental capacity or language barriers) and patients previously randomised to this trial will be excluded from taking part in the research.

### Proposed sample size

Assuming the proportion of patients who experience recurrence following RBL is 30% and following HAL is 15%, the sample size required to detect a difference in recurrence rates with 80% power and 5% significance is 121 individuals per group. In order to account for any between-surgeon variation and loss to follow-up, we propose increasing this to 175 per group. This increase is based on the conservative assumption that there will be 14 surgeons in the trial (one per centre) and intra-class correlation (ICC) of 2.5% in keeping with typical ICCs observed by Ukoumunne [21]. A more likely scenario is that each site will have a minimum of two surgeons, in which case the power to detect this difference is 85%; if there is no between-surgeon variation, the power will be 90%. Because the surgical procedure is well-developed and standardised, intra-class correlation should be virtually zero and the proposed sample size should have closer to 90% power.

The impact of loss to follow up will be minimal for the primary endpoint (haemorrhoidal recurrence at 12 months). Patients who do not complete their 12-month follow-up will have their hospital notes reviewed and their GP will be written to in order to ascertain whether any complications or operative procedures were recorded. The only drop-out expected would be where the patient dies, moves out of the area, or has no traceable patient notes, and we anticipate this would be less than the 5% we have now allowed for in this patient population (a previous study of RBL which used only clinical follow up, reported a 1-year loss to follow up of 10% [22]).

### Interventions

The intervention is either RBL or HAL. Both interventions are established and well documented procedures. Both intervention arms are considered standard care by NICE.

Conventional RBL uses a simple suction device that is applied to each haemorrhoid via a disposable proctoscope. A rubber band is then fired onto the base of the haemorrhoid which constricts the blood supply causing it to become ischaemic before being sloughed approximately 1–2 weeks later. The resultant fibrosis reduces any element of haemorrhoidal prolapse that may have been present. This is a very commonly performed procedure in all SOPCs; figures from an audit of current practice at STH over 20 such procedures are carried out every week. The procedure is a basic surgical skill that all senior staff are familiar with and competent in performing.

HAL uses a proctoscope modified to incorporate a Doppler transducer. This enables accurate detection of the haemorrhoidal arteries feeding the haemorrhoidal cushions. Accurate ligation of the vessels with a suture reduces haemorrhoidal engorgement. When combined with a ‘pexy’ suture, both bleeding and haemorrhoidal prolapse is addressed. All surgeons participating in the trial will ensure the need for a pexy suture is routinely assessed and recorded.

The procedure is simple, uses existing surgical skills and has a short learning curve, with the manufacturers recommending at least 5 mentored cases before independently practising. All surgeons involved in the study will have completed this training and will have carried out over 5 procedures prior to recruiting to the study.

### Safety Assessments

We will collect data on related Adverse Events (AEs) on the Case Report Forms (CRFs). Where these events become Serious Adverse Events (SAEs) they will be reported in accordance with the CTRU’s and the sponsor’s Standard Operating Procedures (SOPs). These SOPs have been developed to comply with guidance from the National Research Ethics Service, which is a subdivision of the National Patient Safety Agency, and Good Clinical Practice (GCP). Site staff will be responsible for reporting SAEs; on identification they will complete an SAE form and send it to the CTRU and ensure that the local Principal Investigator has been informed.

Details of any related AEs will be recorded on the case report forms and participant completed questionnaires and reported periodically to the Sponsor, Data Monitoring Committee (DMEC) and the Trial Steering Committee (TSC). SAEs related to the intervention and unexpected will be reported Sponsor and expedited to the Research Ethics Committee (REC) within 15 days of becoming aware.

### Objectives

The primary research question is does haemorrhoidal artery ligation have a lower recurrence rate than rubber band ligation when used to treat second and third degree haemorrhoids?

The secondary research questions are which of the two procedures is more cost-effective; which is least painful; which has fewest complications and which has the greatest effect on the patients’ quality of life?

### Outcomes

The primary outcome measure is ‘recurrence’, defined as the proportion of patients with recurrent haemorrhoids at 12 months, as derived from a telephone assessment in combination with GP and hospital records.

The trial is a pragmatic design with a dichotomous outcome. As no validated symptom score exists, we have based our definition of recurrence on Shanmugam et al.’s systematic review [6] definition:

1. Cured or improved: Symptom free or mild residual symptoms but not requiring further treatment at the end of study period; or,

2. Unchanged or worse: No symptom improvement and requiring further intervention or suffered complication or deterioration of symptoms.”

This study will simplify Shanmugam's criteria into the following question, asked at 12 months by a research nurse:

‘At the moment, do you feel your symptoms from your haemorrhoids are:

1. Cured or improved compared with before starting treatment; or,

2. Unchanged or worse compared with before starting treatment?’

Any patient who answers ‘1’ but has required further treatment since the initial procedure will be reclassified as ‘2’, identified via hospital records, their consultant, their GP and patient questioning.

Secondary outcome measures:

1. Symptom score (adapted from Nystrom et al. [23])

2. Health related quality of life (using the EuroQol-5D “The EuroQoL group” [24])

3. Continence questionnaire (using the validated Vaizey Incontinence Score [25])

4. Pain score (using a 10 cm visual analogue scale)

5. Surgical complications

6. Need for further treatment including details

7. Clinical appearance of haemorrhoids at proctoscopy following recurrence

8. Health care costs

9. Cost effectiveness

Eligible patients who have given written informed consent to participate in the study will undergo baseline assessment immediately before randomisation (Symptom score, EQ-5D, Continence questionnaire). After their procedure, patients will be asked to complete questionnaires one, seven and twenty-one days after surgery (EQ-5D, Pain score). At six weeks, further data will be collected at the routine follow-up clinic visits (Symptom score, EQ-5D, Pain score, Health and social care resource use questionnaire, Complications review interview, Need for further treatment questionnaire). Finally, one year after their procedure, they will be sent questionnaires (Symptom score, EQ-5D, Continence questionnaire, Health and social care resource use questionnaire, Complications review interview, Need for further treatment questionnaire, Recurrence) and be followed up by telephone. We will measure recurrence (the primary outcome) at 12 months.

### Statistical and Health Economic analyses

Differences in the primary outcome, recurrence of haemorrhoids between the two treatment groups, will be analysed using logistic regression adjusting for sex, age at surgery and history of previous intervention as fixed effect covariates and surgeon as a random effect. This permits the calculation of odds ratios and their confidence intervals for the effect of HAL relative to traditional RBL adjusting for the effects of covariates and the clustering by surgeon. Further detailed analysis of haemorrhoid recurrence will be performed by analysing the length of time to recurrence under a Cox-proportional hazards model adjusting for the same covariates. The secondary outcome of pain, as measured on the visual analogue scale (VAS), with repeated measures will be analysed under a multi-level longitudinal approach adjusting for the same covariates. The secondary outcome of procedural complications elicited during the complications review or from the patient notes at 6 weeks and one year post surgery, will be compared between the two groups at each time point using Poisson regression which accounts for the essentially random nature with which complications arise, although care will be taken to note whether there is any clustering by surgeon. The emphasis of all analyses will be on estimating effect sizes of HAL surgery on recurrence rates and other outcomes in comparison to standard treatment with RBL, and as such appropriate confidence intervals will be reported for all estimates.

We will collect data as part of the trial that will allow us to conduct a full economic evaluation. The main economic analysis will focus on estimating the incremental cost per quality adjusted life year (QALY) of HAL versus RBL over the 12 month follow up period of the trial in: a) patients with new haemorrhoids; and, b) patients with recurrence following RBL. We will also present results in terms of the incremental cost per recurrence avoided.

The time horizon compares to 12 months used in an evaluation of SH versus RBL in patients with grade II haemorrhoids conducted alongside a pilot clinical trial [26] and 3 years in a modelling study comparing SH and OH [2]. The former study recommended larger trials and longer follow up. Since it is likely that both surgical complications and recurrence rates will differ at 12 months we will also use decision modelling to extrapolate beyond the trial outcomes. We will draw on and develop as appropriate the model reported in the HTA report [2]. This model is also being considered for adaptation alongside a model that includes RBL, by those conducting the eTHoS trial (HTA 08/24/02) of stapled haemorrhoidopexy (SH) vs. traditional haemorrhoidectomy (OH). We will liaise with the eTHoS project team to ensure consistency, where appropriate, in our approaches to model adaptation. This will include issues around both model structure and time horizon, as well as parameter values where these are common to both decision problems. This will also avoid unnecessary duplication of workload, particularly in relation to reviewing.

Patients will be asked to complete the EQ-5D instrument at baseline and 1 day, 7 days, 21 days, 6 weeks and 12 months following the treatment. The UK population tariffs will then be used to calculate QALYs for each patient. EQ-5D has been applied in previous studies in this area [26] and appears to be sensitive to changes in patient outcomes. Pain is likely to be one of the main symptoms in which we might expect the treatments to differ and this is well reflected in the EQ-5D instrument.

In addition, we will explore the relationship between EQ-5D values and specific complications and symptoms, such as relapse, incontinence and pain. We will also consider the relationship between EQ-5D and the combined symptom severity score. These explorations may prove valuable to future modelling studies in this area as there is evidence that previous studies have been hampered by limited utility data. Indeed, a specific recommendation of a recent HTA study was “that further research should include RCTs which collect a generic HRQoL measure such as the EQ-5D or SF-36 at follow-up times close to the procedure and, in the long term, calculate an estimate of preference-based utility. Baseline data from a trial of this kind would also provide a better estimate of HRQoL and utility of patients with symptoms.” (p.88 HTA report [2])

We will use appropriate statistical techniques to reflect skewness, repeated measures from individual patients and the clustering of the patients, by surgeon and within different centres. Parameter uncertainty will be fully reflected in the estimates by generating a cost effectiveness acceptability curve. We will also consider other forms of sensitivity analysis to reflect further sources of uncertainty.

### Ethical approval

This research has been approved by South Yorkshire REC.

## Discussion

Alongside the eTHoS trial (HTA 08/24/02) the findings of the research will help inform future practice for the treatment of grade II and III haemorrhoids. The results will enable clinicians to provide patients with up-to-date, robust information so that they can make an informed choice of the treatment option most appropriate to their individual needs. Our findings will be disseminated through the Association of Coloproctology of Great Britain and Ireland.

## Abbreviations

AE: Adverse event; CRF: Case report form; CTRU: Clinical trials research unit; DMEC: Data monitoring and ethics committee; GCP: Good clinical practice; GP: General practitioner; HAL: Haemorrhoidal artery ligation; HRQoL: Health related quality of life; NICE: National Institute for Health and Clinical Excellence; NHS: National health service; OH: Open haemorrhoidectomy; QALY: Quality adjusted life year; RBL: Rubber band ligation; RCT: Randomised control trial; SAE: Serious adverse event; ScHARR: School of health and related research; SH: Stapled haemorrhoidopexy; SOP: Standard operating procedure; SOPC: Surgical outpatient clinic; TSC: Trial Steering Committee; VAS: Visual analogue scale.

## Competing interests

The authors have no competing interests relating to this research or publication.

## Authors’ contributions

SB, JT, DH, AW, AJW, MB and NS worked on the conception and design of the study and secured the funding for the research. SB is the lead investigator. All authors contributed to the writing of the protocol.

## Pre-publication history

The pre-publication history for this paper can be accessed here:

http://www.biomedcentral.com/1471-230X/12/153/prepub
